# *Candida albicans* brain abscesses in an injection drug user patient: a case report

**DOI:** 10.1186/1756-0500-7-837

**Published:** 2014-11-25

**Authors:** Nélia Neves, Lurdes Santos, Carina Reis, António Sarmento

**Affiliations:** Infectious Diseases Department, Centro Hospitalar São João and Faculty of Medicine of Porto University, Porto, Portugal; Neuroradiology Department, Centro Hospitalar São João and Faculty of Medicine of Porto University, Porto, Portugal

**Keywords:** Brain abscess, Candida albicans, Invasive candidiasis, Injection drug user, Diabetes

## Abstract

**Background:**

Fungal brain abscess is an uncommon disease, mostly associated with immunocompromised states and poorly controlled diabetes. Its incidence, however, is rising as a result of the increasing use of immunosuppressive agents, corticosteroids and broad-spectrum antimicrobial therapy. *Candida* species have emerged as the most prevalent etiologic agents of brain abscesses in autopsy studies.

**Case presentation:**

A 46-year-old male with a history of injection drug abuse, chronic hepatitis C and diabetes mellitus presented to the Emergency Department of our hospital following a generalized tonic-clonic seizure without recovery of mental status. On admission, the patient was in coma, febrile, with severe acidemia with respiratory and metabolic acidosis, requiring invasive mechanical ventilation. Brain imaging revealed multiple ring-enhancing lesions with oedema and mass effect. Microbiologic studies, including cerebrospinal fluid, blood, sputum and urine cultures, were all negative. A stereotactic brain biopsy was performed and culture of brain specimens revealed *Candida albicans*. The patient was successfully treated with fluconazole therapy for 48 weeks presenting a good clinical response and a complete radiological resolution of brain abscesses.

**Conclusion:**

Despite advances in diagnostic and therapeutic procedures, fungal brain abscess remains a life-threatening disease with a poor outcome. Successful treatment requires an early diagnosis and usually a combined medical and surgical approach. A long-term antibiotic regimen is a cornerstone of fungal brain abscesses treatment, with the endpoint determined by clinical and neuroimaging response. The authors report an uncommon case of successfully treated *Candida albicans* brain abscesses with anti-fungal therapy consisting of fluconazole alone. This case illustrates the importance of early recognition of predisposing factors and multidisciplinary approach in timely therapeutic intervention, in order to prevent neurologic sequelae and improve the outcome of the patients with this severe and challenging form of central nervous system infection.

## Background

Brain abscesses are focal and purulent infections of brain parenchyma and may be a result of a local process or secondary to a distant primary infection. Despite new advances in the management of brain abscess, mortality rate is high and neurologic sequelae are reported in 20 to 70% of the patients who survives a brain abscess [1].

Brain lesions in injection drug user (IDU) patients present an extensive differential diagnosis including both infectious and noninfectious causes. The clinical presentation of brain abscess in IDUs is the same as in nonusers and depends on the size and location of the lesion. Common symptoms and signs include headache, altered mental status, focal neurologic findings and fever; seizures may occur in up to 40% of the patients [1–3].

The pathogens responsible for brain abscesses depend on the source of infection and patient specific risk factors. Fungal brain abscesses are uncommon diseases but their incidence have increased as a result of the prevalent use of immunossupressive agents, corticosteroids and broad-spectrum antimicrobial therapy [1, 4–6]. Invasive fungal diseases are associated with immunocompromised states, including malignancy and neutropenia, poorly controlled diabetes and central venous catheterization [1, 4, 7]. In immunocompromised individuals, the pathogens frequently responsible for invasive fungal infections are *Candida* spp, *Aspergillus* spp, *Cryptococcus* spp and Mucorales [7, 8]. *Candida* spp. have emerged as the most prevalent etiologic agents of brain abscesses in autopsy studies [1], and many reports and cases series describing *Candida* brain abscesses have been published in literature [4, 8–17]. In IDUs, the most common cause of CNS disease is endocarditis, which has also been associated to the most severe complications, as brain abscess, meningitis and brain hemorrhage [2, 18, 19]. The authors report a case of cerebral abscesses due to *Candida albicans* in an IDU and diabetic patient who was admitted to our hospital following a generalized seizure, in which endocarditis was excluded.

## Case presentation

A 46-year-old male with a history of chronic hepatitis C and injection drug use presented to the Emergency Department of our Hospital following a generalized tonic-clonic seizure without recovery of mental status. He also had a history of heavy alcohol intake with recurrent episodes of pancreatitis and occasional epileptic fits, starting seven years prior, for which the patient did not take any medication. He was born in Portugal and had no travel exposure.

One month before this presentation, the patient had an episode of acute pancreatitis and was admitted to the Surgical Department of our Hospital. At that time a type-2 diabetes mellitus was diagnosed and insulin therapy was started. During his stay in the hospital, he developed alteration in mental status with increasing agitation for which a head CT scan was performed that showed signs of left otomastoiditis, without brain lesions. A lumbar puncture was also performed with normal cerebrospinal fluid (CSF). Ear-nose-throat (ENT) specialist consultation confirmed the diagnosis of acute otitis media and left otomastoiditis, and the patient was started on therapy with ceftriaxone 2 g qd. His neurologic changes were attributed to withdrawal symptoms and he was discharged on day 20 with referral for ENT and surgical review. Following discharge, he became confused and developed ataxia and blurred vision. Two weeks later he had a new episode of generalized tonic-clonic seizure without recovery of mental status and was readmitted to our Hospital.

On arrival to the emergency department, the patient was in coma, with a score of 3 (E = 1, M = 1, V = 1) on Glasgow Coma Scale (GCS). He was febrile (temperature was 38.0°C), hemodynamically stable (mean arterial pressure was 65 mmHg and heart rate was 80 beats per minute), with a normal capillary blood glucose level (110 mg/dL). The respiratory rate was 20 breaths per minute and peripheral oxygen saturation was 94%, at room air. He presented skin lesions suggestive of intravenous injections on his arms. Chest auscultation revealed rhonchi bilaterally, with no other abnormalities on physical examination. Arterial blood gas (ABG) measurement showed severe acidemia with respiratory and metabolic acidosis (pH - 7.015, PaO_2_ - 96.9 mmHg, PaCO_2_ - 64.9 mmHg HCO_3_^−^ - 16.2 mmol/L) and a high lactate level of 12.07 mmol/L (normal, <1 mmol/L). Posteroanterior chest radiography was clear. The patient was intubated and invasive mechanical ventilation was started on emergency room (ER).

The white blood cell (WBC) count was 15.9 × 10^9^/L, with 58% lymphocytes. Laboratory studies showed rabdomyolisis and a creatinine of 15.1 mg/L (normal, 8.0 – 13.0 mg/L) with normal electrolytes levels. Amylase and lipase were elevated two times the upper limit of normal; liver function tests were normal, except for a slight elevation of γ-glutamyltransferase (γ-GT), and C-reactive protein (CRP) was normal (Table 1). Toxicology screen was positive for opioids and benzodiazepines. Urinary antigen detection for *Streptococcus pneumonia* and *Legionella pneumophila* was negative and specimens of blood, sputum and urine were cultured. Testing for human immunodeficiency virus (HIV) was negative.

**Table 1:** **Serum analyses on admission**

Serum analyses		Reference intervals
**Haemoglobin**	14.6 g/dL	13.0 – 18.0 g/dL
**Leucocytes**	**15.9 x 10** ^**9**^ /**L**	4.0 – 11.0 x 10^9^ /L
**Neutrophils**	5.18 x 10^9^ /L (**32**%)	53.8 – 70%
**Lymphocytes**	9.37 x 10^9^ /L (**58**%)	22.6 – 36.6%
**Platelets**	257 x 10^9^ /L	150 – 400 x 10^9^ /L
**Lactate dehydrogenase,** **LDH**	207 IU/L	135 – 225 IU/L
**Creatine kinase,** **CK**	189 IU/L	10 – 172 IU/L
**Myoglobin**	1073.4 ng/mL	< 146 ng/mL
**Creatinine**	**15.1 mg**/**L**	8.0 – 13.0 mg/L
**Urea**	0.41 g/L	0.10 – 0.50 g/L
**Calcium**	5.0 mEq/L	4.2 – 5.1 mEq/L
**Magnesium**	1.62 mEq/L	1.55 – 2.05 mEq/L
**Sodium**	138 mEq/L	135 – 147 mEq/L
**Potassium**	3.7 mEq/L	3.5 – 5.1 mEq/L
**Alanine transaminase,** **ALT**	32 IU/L	10 – 37 IU/L
**Aspartate transaminase,** **AST**	38 IU/L	10 – 37 IU/L
**Alkaline phosphatase,** **AP**	89 IU/L	30 – 120 IU/L
**γ**-**glutamyltransferase,** **γ**-**GT**	**62 IU**/**L**	10 – 49 IU/L
**Bilirubin** **(Total/** **Direct)**	5.8 / 2.3 mg/L	< 12 / <4 mg/L
**Amylase**	**116 IU**/**L**	22 – 80 IU/L
**Lipase**	**118 IU**/**L**	7 – 60 IU/L
**C** **-** **reactive protein,** **CRP**	1.5 mg/L	<3 mg/L

Patient’ serum analyses, including haematology and biochemical values, on admission to the emergency department after a generalized seizure without recovery of mental status. Bold text highlights the values outside the normal range.

**Figure 1:**
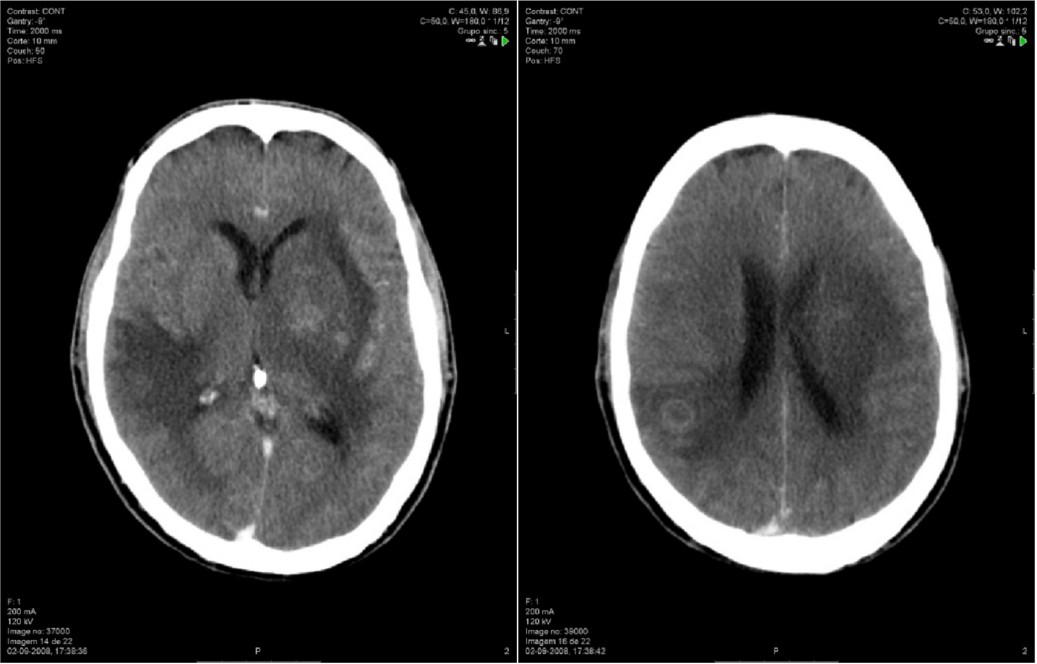
**Head computed tomography** **(CT)** **scan on admission.** Showed heterogeneous densities involving basal ganglia particularly in the left side and right temporo-parietal region, with mass effect. Post contrast images depicted ring-enhancing lesions.

Head computed tomography (CT) scan showed multiple contrast ring enhancing lesions, in left basal ganglia and right subcortical temporo-parietal region, with oedema and mass effect (Figure 1). A lumbar puncture was performed and analysis of the CSF showed normal levels of white-cell count, proteins and glucose (2 cels/uL, 0.10 g/L and 0.55 g/L, respectively); CSF was sent for culture, molecular tests and pathological examination. Electroencephalogram (EEG) showed non-specific encephalopathy with epileptiform activity in right frontal region. Intravenous (IV) meropenem 2 g tid was started empirically on ER for suspected bacterial cerebral abscesses, and adjunctive therapy with mannitol and dexamethasone was added. The patient was transferred to the Infectious Diseases Intensive Care Unit (ICU) with these focal brain lesions with mass effect of unknown etiology.

**Figure 2:**
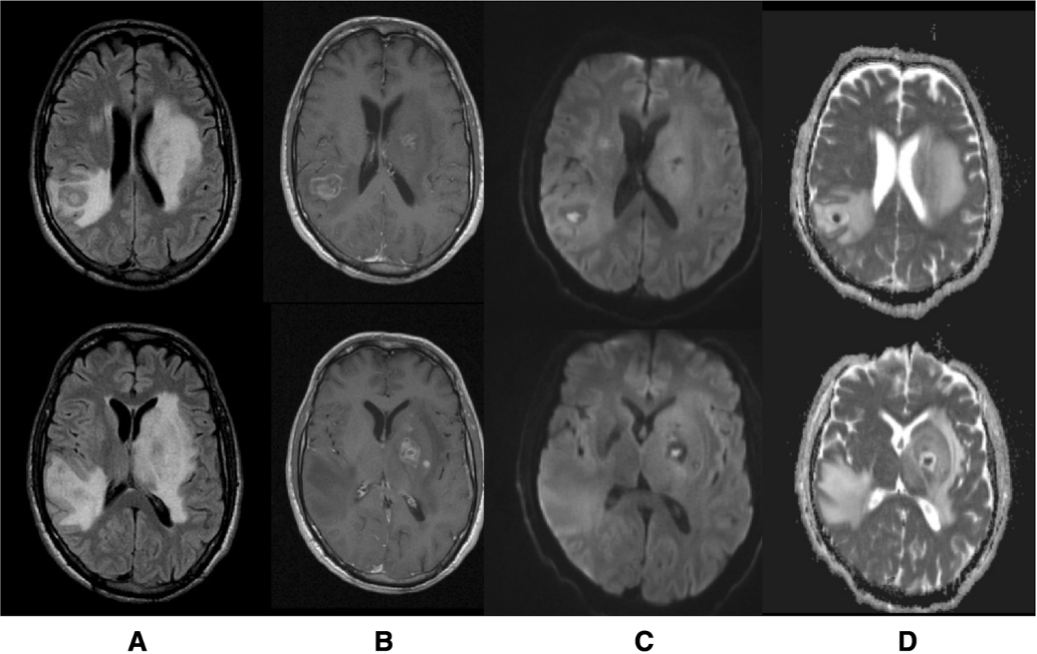
**Brain magnetic resonance imaging** **(MRI)** **on day 2.** Showed multiple rounded expansive lesions in basal ganglia region predominantly on the left side and in subcortical right parietal region with prominent oedema, resulting in modelling of lateral ventricles, slight deviation of middle line to the right side and reduction of sulci dimensions (**A:** T2 flair). These lesions present a post gadolinium multilayered ring enhancement pattern (**B:** T1 post gadolinium) and central core with restrition of water molecules diffusibility (**C**: DWI, **D:** ADC map).

After admission to the ICU, a transesophageal echocardiogram was performed and ruled out signs of endocarditis. Ophthalmology specialist consultation confirmed the absence of ocular involvement and chest-abdomen CT ruled out other possible foci. Brain magnetic resonance imaging (MRI) revealed multiple lesions involving basal ganglia bilaterally and subcortical right parietal white matter, with surrounding oedema, resulting in modelling of lateral ventricles, slight midline deviation and reduction of basal cisterns and sulci dimensions (Figure 2). These lesions were heterogeneous, some with multilayered signal differences and double-ring enhancement, and smaller ones presenting single layer ring enhancement in post gadolinium images. All lesions showed a core with restriction diffusibility to water molecules movement on diffusion weighted imaging (DWI, Figure 2). No haemorrhage was seen. These imagiological findings were consistent with brain abscesses, most probably fungal or pyogenic, the latter less likely due to the double-layer pattern enhancement. On day 2, empiric anti-fungal therapy with IV fluconazole 800 mg qd was added to the therapeutic regimen.

CSF studies including polymerase chain reaction (PCR) assay for toxoplasma, Epstein-Barr virus (EBV) and *Mycobacterium tuberculosis* were all negative, as was CSF culture. On CSF pathological examination no neoplastic cells were found. Blood, sputum and urine cultures were also negative. Neurosurgery consultation was called and a stereotactic brain biopsy was performed in order to highlight the causative pathogen. Histopathological examination of brain specimens showed a mononuclear cell infiltrate, with no evidence of tumor. Acid fast bacilli (AFB) smear of brain specimens was negative and PCR assay for *Toxoplasma*, *Mycobacterium tuberculosis*, *Candida albicans* and *Aspergillus* were also negative. Microbiologic culture of brain specimens revealed *Candida albicans*.

**Figure 3:**
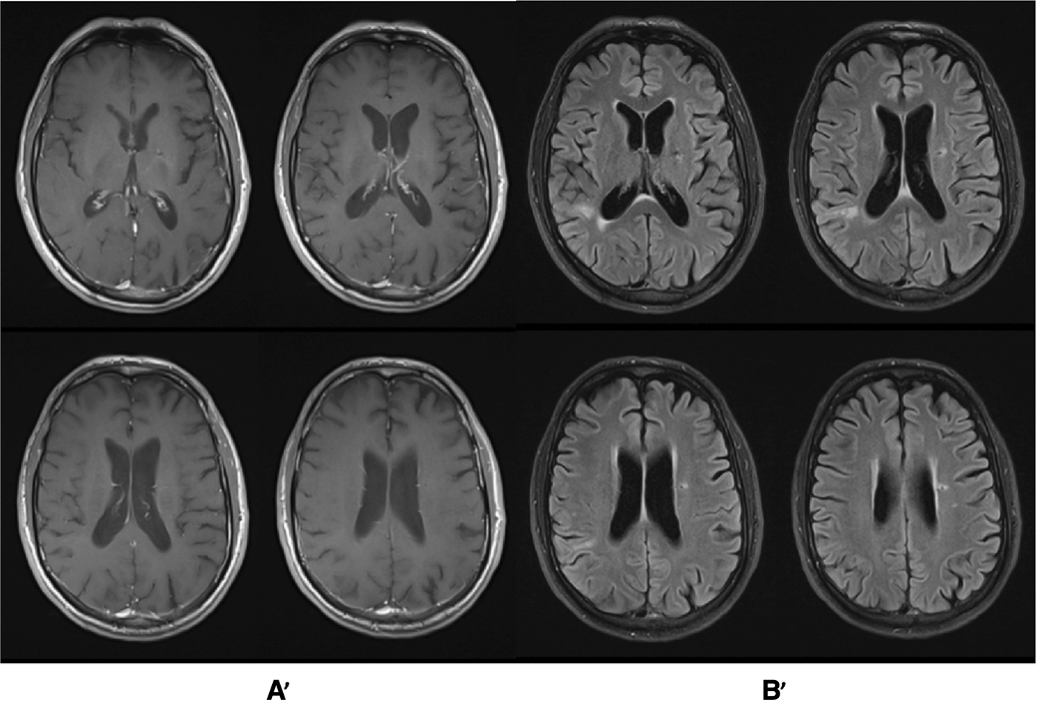
**Follow**-**up brain magnetic resonance imaging** **(MRI).** After antifungal treatment with fluconazole for 49 weeks, showing a complete resolution of brain abscesses with global atrophy. (**A**’: T1 post gadolinium; **B**’: T2 flair).

Antimicrobial therapy was maintained with fluconazole and meropenem was withdrawn. The patient was extubated on day 12 and sedation was weaned off with recovery of his conscience level. Brain MRI was repeated three weeks after and showed radiological improvement with reduction of surrounding oedema but with persistent enhancing lesions. The patient recovered without neurologic disability and had a good radiological response on subsequent MRI. He was discharged home after two months of hospitalization under anti-fungal therapy consisting of oral fluconazole 400 mg bid and anticonvulsant drugs. He did well on outpatient follow-up, without new episodes of seizures and neurologic sequelae. Fluconazole therapy was maintained for 49 weeks when a complete resolution of brain abscesses was seen on follow-up brain MRI (Figure 3).

## Conclusions

Brains abscesses most commonly result from a contiguous spread of infection from paranasal sinusitis, otogenic or odontogenic sources, or result from haematogenous spread from distant locations, including endocarditis, lung abscesses, intra-abdominal or genitourinary source. Other risk factors include penetrating head trauma, neurosurgical procedure or injection drug use. Brain abscesses are frequently polymicrobial, in up to 60% of the cases, and anaerobic bacteria are often isolated; however, its epidemiology has changed with the increasing incidence in immunocompromised patients and the decreasing occurrence related to sinusitis and otitis [10, 20, 21].

CNS *Candida* infections in adults can occur as a manifestation of disseminated candidiasis. Meningitis is the most common presentation of CNS *Candida* infections, but other neuropathologic manifestations includes microabscesses (<3 mm), macroabscesses, noncaseating granulomas, and diffuse glial nodules [1, 6, 22–24]. In an extensive study of necropsies that included 2107 cases, CNS infection was observed in 92 patients (4%); in most of these, the infection took the form of disseminated microabscesses usually found in the gray-white junction, basal ganglia, and cerebellum [22].

In patients presenting with brain abscesses, invasive candidiasis should be considered if there is a history of immunosuppression, malignancy, neutropenia, chronic granulomatous disease, diabetes mellitus, thermal injuries, intravenous drug use, central venous catheter (CVC) or prolonged antimicrobial therapy, particularly if there was a poor response to antibacterial agents [4, 25–27]. Some cases of *Candida* brain abscesses have been described in HIV patients [11–13] and also in premature infants, since prematurity itself contributes to immunosuppression and it is often associated with prolonged hospitalization, CVC placement and antibiotic use [4, 26, 27].

In this particular patient, possible risk factors for fungal brain abscess which were identified included injection drug abuse and poorly controlled diabetes. We believe that hematogenous spread from IV injection was the most likely source of infection, since other possible sources of brain abscesses were ruled out, including endocarditis, paranasal sinusitis or otogenic infection. Other important immunocompromised conditions, as human immunodeficiency virus (HIV) infection, were also excluded in our patient.

Diabetes has been described in literature as a frequent predisposing condition for fungal brain abscess [1, 7]. Although diabetic patients are characterized by an increased susceptibility to infection possibly associated with impaired innate and acquired immunity [28–30], the role of diabetes as immunosuppressive condition in this particular case is uncertain. In our patient, diabetes was probably secondary to pancreatitis and it is not completely known if secondary diabetes is characterized by the disturbance in immune response observed in type 2 and type 1 diabetes, since the pathogenesis of these conditions are different. On the other hand, some microorganisms may present specific mechanisms that can lead to an increased prevalence of infection in diabetic patients, as an increased adherence to host cells which has been described for *Candida albicans* [31].

Laboratory and imaging studies may be a clue to the etiologic diagnosis, since the clinical presentation of fungal brain abscesses may be similar to bacterial abscess. In a recent review of literature of *Candida* brain abscess case reports, blood cultures revealed candidemia in only 55% of the cases reviewed and lumbar puncture yielded *Candida* growth in only 23% of cases [4]. Thus, the diagnosis of candidal brain abscess cannot be ruled out in the absence of candidemia and abnormal CSF, because blood cultures and CSF studies have variable results and may not be helpful.

Neuroimaging studies, including CT and MRI, are important diagnostic tools for differential diagnosis of brain lesions and also in monitoring the response to therapy. Over the past decades, advances in neuroimaging, particularly diffusion-weighted imaging (DWI), have improved the ability to differentiate brain abscesses from other ring-enhancing lesions. In this case, the post contrast ring enhancement together with a central core of restricted diffusion indicates increased viscosity and inflammatory response, which is compatible with brain abscess [32, 33]. In addition, the multilayered ring pattern on MRI was strongly suggestive of fungal lesions and is not typically seen in pyogenic abscesses nor in tuberculosis [34–37]. Other diagnosis as toxoplasmosis and neoplastic conditions were less likely due to the central diffusion restriction. Therefore, MRI studies provided a more specific guidance in narrowing diagnostic possibilities and the presumed fungal etiology allowed early administration of antifungal chemotherapy in our patient.

Despite of modern imaging studies now available, the definite diagnosis of brain lesions is frequently difficult to establish and invasive strategies, as stereotactic brain biopsy, may be required. Surgical approaches, including drainage or lesion excision, may be both diagnostic and therapeutic in order to obtain source control. Improved microbiologic culture techniques have an important impact on the awareness of microbiological identification in brains specimens. In this case, culture of brain specimens obtained by stereotactic biopsy allowed the isolation of the responsible agent, while other microbiologic exams performed were negative, including blood cultures, CSF culture and molecular testing.

Given the frequent polymicrobial nature of brain abscesses, broad-spectrum empiric antimicrobial therapy is indicated. The regimen choice depends on risk factors for specific organisms, including medical history, immune status and location of primary infection. In immunocompetent patients, initial coverage with a third-generation cephalosporin, as ceftriaxone or cefotaxime, associated with metronidazole is recommended [1, 7, 38]. Alternatively, ceftazidime, cefepime or a carbapenem can be used, particularly if nosocomial pathogens as *Pseudomonas aeruginosa* are suspected [1]. Vancomycin should be added empirically in patients with post-neurosurgical brain abscesses or after cranial trauma, in patients with endocarditis or Methicillin-resistant *Staphylocccus aureus* (MRSA) bacteremia and whenever *S. aureus* infection is suspected pending culture results [1, 7]. Once a causative pathogen is isolated, antimicrobial therapy should be tailored. In this particular patient, vancomycin could have been initially added considering his injection drug use history and major risk factors for MRSA colonization, including recent hospitalization and previous exposure to antibiotic therapy. Addition of amphotericin B should be considered in neutropenic and post-transplant patients to cover pathogenic fungi, such as *Aspergillus* spp., *Cryptococcus* spp. and Mucorales. If *Nocardia* spp. is suspected trimethoprim/sulfamethoxazole should be added to the empiric regimen [21, 39].

The mainstay of medical therapy for candidal brain abscess is an amphotericin B preparation. Although amphotericin B is considered the first-line agent for most CNS fungal infections, it has a large molecular weight and poor penetration of the blood–brain barrier (BBB). For this reason, 5-flucytosine should be added to amphotericin B for the treatment of candidal, cryptococcal and *Aspergillus* CNS infections. The current European Society of Clinical Microbiology and Infectious Diseases (ESCMID) guideline recommend treatment of CNS candidiasis with IV amphotericin B plus 5-flucytosine for several weeks or with fluconazole if isolate is susceptible, however no strong recommendation can be given due to lack of data [6]. Voriconazole, a second-generation triazole, exhibits a broad spectrum of activity with the exception of the Zygomycetes. It is the drug of choice for invasive aspergillosis and achieves satisfactory levels in brain tissue, however published data on its use in CNS candidiasis are sparse [6, 23, 40–42].

Optimal treatment should also include management of risk factors for fungal disease, such as hyperglycemia and dosage reduction or elimination of immunosuppressive agents when appropriate [1, 43]. In this case, fluconazole was started empirically considering several facts: fluconazole is a possible agent of choice for the treatment of disseminated candidiasis in non-neutropenic and haemodinamically stable patients [6, 40]; it has good CNS penetration, and the majority of *Candida albicans* isolates in our hospital is sensitive to fluconazole. Although the isolated pathogen was covered by fluconazole, a broader-spectrum empiric antifungal therapy would be desirable for a possible fungal brain abscess.

Duration of antimicrobial therapy for brain abscesses remains unclear; it should be individualized and guided by clinical evolution and neuroimaging response. Due to paucity of convincing data supporting optimal duration of medical treatment, parenteral antimicrobial therapy has been traditionally given for at least six to eight weeks, followed by oral antimicrobial therapy [1, 7, 44]. This patient was successfully treated with medical therapy alone consisting of IV fluconazole for nine weeks followed by forty weeks of oral fluconazole with a complete radiological resolution of the abscesses.

Treatment of fungal brain abscesses remains a challenge and the optimal therapy usually requires a combined medical and surgical approach. Neurosurgery and Radiology consultation should be obtained to address stereotactic aspiration using CT or MRI guidance, surgical drainage or complete excision of the abscess. The indication for surgery is based on patient factors, location and size of brain lesions. Candidates for medical therapy alone include patients with multiple abscesses, small size (<2.5 cm), difficult locations or patients with a severe surgery risk associated [7, 44–46]. Surgical excision is less frequently performed because of the development of stereotactic procedures, but it may be required in multiloculated abscesses, abscesses containing gas or which fail to resolve [1].

Despite the introduction of stereotactic brain biopsy, aspiration techniques and new antifungal agents, fungal brain abscess remains a life-threatening disease with a poor treatment outcome [4, 9, 47]. Poor prognostic factors in brain abscess include the presence of predisposing conditions, other comorbidities, a poor Glasgow Coma Scale score and brain abscess complicated by intraventricular rupture [1, 48, 49].

A multidisciplinary approach is required for management of the patient with brain abscess and should involve neurosurgery, infectious diseases, neuroradiology and critical care medicine. Successful treatment of *Candida* cerebral abscess requires an early diagnosis, based on high index of suspicion and specific diagnostic procedures, and a long-term antibiotic treatment, in which the endpoint should be determined by clinical and radiographic resolution of the abscess. Early recognition of predisposing factors and a multidisciplinary approach is important for improving the outcome of the patients with this severe and challenging form of CNS infection.

## Consent

Written informed consent was obtained from the patient for publication of this Case report and any accompanying images. A copy of the written consent is available for review by the Editor of this journal.

## Abbreviations

ABG: Arterial blood gas
ADC: Apparent diffusion coefficient
AFB: Acid fast bacilli
BBB: Blood–brain barrier
CNS: Central nervous system
CRP: C-reactive protein
CSF: Cerebrospinal fluid
CT: Computed tomography
DWI: Diffusion weighted imaging
EBV: Epstein-Barr virus
EEG: Electroencephalogram
ENT: Ear-nose-throat
ER: Emergency room
ESCMID: European Society of Clinical Microbiology and Infectious Diseases
GCS: Glasgow coma scale
γ-GT: γ-glutamyltransferase
HIV: Human immunodeficiency virus
IDU: Injection drug user
IV: Intravenous
MRI: Magnetic resonance imaging
MRSA: Methicillin-resistant *Staphylocccus aureus*
PCR: Polymerase chain reaction
WBC: White blood cell.
